# How To Build a Bone: PHOSPHO1, Biomineralization, and Beyond

**DOI:** 10.1002/jbm4.10202

**Published:** 2019-07-07

**Authors:** Scott Dillon, Katherine A Staines, José Luis Millán, Colin Farquharson

**Affiliations:** ^1^ The Roslin Institute and Royal (Dick) School of Veterinary Studies University of Edinburgh, Easter Bush Midlothian UK; ^2^ School of Applied Sciences Edinburgh Napier University Edinburgh UK; ^3^ Sanford Burnham Prebys Medical Discovery Institute, La Jolla CA USA

**Keywords:** PHOSPHO1, PHOSPHOCHOLINE, INORGANIC PHOSPHATE, BIOMINERALIZATION, MATRIX VESICLE

## Abstract

Since its characterization two decades ago, the phosphatase PHOSPHO1 has been the subject of an increasing focus of research. This work has elucidated PHOSPHO1's central role in the biomineralization of bone and other hard tissues, but has also implicated the enzyme in other biological processes in health and disease. During mineralization PHOSPHO1 liberates inorganic phosphate (P_i_) to be incorporated into the mineral phase through hydrolysis of its substrates phosphocholine (PCho) and phosphoethanolamine (PEA). Localization of PHOSPHO1 within matrix vesicles allows accumulation of P_i_ within a protected environment where mineral crystals may nucleate and subsequently invade the organic collagenous scaffold. Here, we examine the evidence for this process, first discussing the discovery and characterization of PHOSPHO1, before considering experimental evidence for its canonical role in matrix vesicle–mediated biomineralization. We also contemplate roles for PHOSPHO1 in disorders of dysregulated mineralization such as vascular calcification, along with emerging evidence of its activity in other systems including choline synthesis and homeostasis, and energy metabolism. © 2019 The Authors. *JBMR Plus* published by Wiley Periodicals, Inc. on behalf of American Society for Bone and Mineral Research.

## Introduction

Biomineralization of the skeleton is a fundamental process indispensable for health and wellbeing throughout life. The vertebrate skeleton is a complex organ that performs varied and diverse functions encompassing its action as a biomechanical and protective scaffold in conjunction with the musculature, its role in calcium and phosphate ion homeostasis, and recent evidence demonstrating its capacity as an endocrine organ involved with energy homeostasis.^1^ At the level of molecular constituents bone is composed of a combination of inorganic mineral, type I collagen, noncollagenous proteins (NCPs), and water, arranged into an extremely ordered hierarchical structure.^2^ The fine details of this architecture remain controversial at the nanostructural level,^3^ particularly with respect to differences between embryonic and mature tissue; however, the mineral phase has come to be regarded as a poorly‐crystalline substituted hydroxyapatite phase, mainly composed of calcium phosphate.

Biomineralization of the skeleton therefore requires generation and manipulation of calcium and phosphate ions on a massive scale. The biological mechanisms through which ions are liberated, contained, and targeted to the developing collagenous framework, and ultimately nucleate mineral in a controlled manner, remain the subject of intense research. Phosphatase enzymes are essential in promoting biomineralization as one pathway through which inorganic phosphate (P_i_) may be liberated from biological molecules. These include enzymes with a canonical role in bone development such as tissue nonspecific alkaline phosphatase (TNAP). Although TNAP activity has long been implicated in the mineralization process, it is now recognized that TNAP is only one component and the full story requires a more complex biochemical system.

Orphan phosphatase 1 (PHOSPHO1; EC 3.1.3.75; encoded by the *Phospho1* gene in the mouse and the *PHOSPHO1* gene in humans) has been an increasing focus of research in this field over the last 20 years and now holds an established function in biomineralization. Here we review the scientific literature on the characterization, localization, regulation, and activity of PHOSPHO1 with respect to its role in physiological and pathological biomineralization, along with potential contributions to other biological processes.

## Characterization, Protein Structure, and Substrate Specificity

PHOSPHO1 (originally known as 3X11A) was initially cloned from chick hypertrophic growth plate chondrocytes, in which a fivefold increase in expression was observed in comparison to resting or proliferative chondrocytes.^4^ This distinct upregulation was also found be approximately 100‐fold higher than in non‐chondrogenic tissues. Although the specific function of this novel protein was at this stage unknown, sequencing of the 3X11A RNA transcript derived from differential display analysis revealed two partially conserved domains that bore sequence similarity to the phosphotransferase enzyme superfamily, including the ATPases and various phosphatases catalyzing P_i_ liberation from a range of phosphomonoester substrates.^4^ Primers designed against these motifs succeeded in transcribing a 537‐bp fragment from RNA isolated from the human osteosarcoma SaOS‐2 cell line, demonstrating 69% similarity to the chicken sequence.^5^ These fragments were identified as corresponding to several previously unattributed EST transcripts from both the human and mouse *Phospho1* gene, with a high degree of conservation at both the gene and protein level between these two mammalian species. Chromosomal mapping led to the conclusion that the *Phospho1* gene was indeed conserved between the bird and mouse genomes, located within a region of conserved synteny on the HSA17 and GGA27 chromosomes, respectively.^5^

Stewart and colleagues^6^ used homology modeling to identify three conserved peptide motifs composing the active site, certifying PHOSPHO1's membership of the Mg^2+^‐dependent haloacid dehalogenase (HAD) superfamily. These included two characteristic aspartic acid residues in motif 1 that coordinate the catalytic Mg^2+^ ion in this family, along with other markers such as the hydrophobic amino acid residues within motif 2. Along with identification of orthology between PHOSPHO1 in rat and the previously identified species, substantial homology between the amino acid sequences of PHOSPHO1 and proteins in the fruit fly was also found, along with several plant species including the LePS2 phosphatases in the tomato plant involved with phosphate homeostasis.^7^, ^8^, ^9^

A PHOSPHO1 model protein structure was developed, based upon the crystal structure of the phosphoserine phosphatases (PSPs).^6^, ^10^ This model revealed conservation of residues complexing the substrate phosphate group; however, those residues that confer substrate specificity to phosphoserine in the PSPs are absent. Roberts and colleagues^11^ purified recombinant human PHOSPHO1 by amplification of a curtailed cDNA transcript from the SaOS‐2 osteosarcoma cell line, and continuous spectrophotometric phosphate assays with several potential phosphomonoester substrates revealed PHOSPHO1's specific activity toward the metabolites phosphoethanolamine (PEA) and phosphocholine (PCho), with highest activity between pH 6.0 to 7.2. Disruption of the protein active site using site‐directed mutagenesis decreased PEA and PCho hydrolysis dramatically to an undetectable level in some mutants.^12^

## Bone Biology and Mineralization

The ubiquitous phosphatase TNAP has a long‐established role during skeletal biomineralization. Mutations in the *ALPL* gene are associated with several skeletal disorders in humans including various forms of hypophosphatasia.^13^, ^14^, ^15^ Genetic ablation of TNAP in a mouse model induced a phenotype mimicking infantile hypophosphatasia with severe skeletal abnormalities,^16^, ^17^ including those in dental and craniofacial mineralization and morphology.^18^, ^19^ Detailed ultrastructural examination of these mice revealed that although hypomineralization of the skeleton was evident, hydroxyapatite mineral crystals were generated as normal.^20^ The hypomineralized phenotype was therefore attributed to the inability of the mineral phase to propagate in the absence of TNAP's hydrolysis of the potent mineralization inhibitor pyrophosphate (PP_i_).^20^, ^21^

These observations led to the hypothesis that another phosphatase was active during skeletal biomineralization with PHOSPHO1 a strong candidate to fulfill this role. Following the observation of *PHOSPHO1* upregulation in mineralizing hypertrophic chondrocytes,^4^ immunohistochemistry revealed localization of the protein to these cells in the chick growth plate, along with active bone surfaces at the ossification front and in the trabecular compartment.^22^ Whole‐mount in situ hybridization in the embryonic chick lower limb furthermore demonstrated expression of *PHOSPHO1* restricted to the developing bones during ossification.^23^ In vivo suppression of PHOSPHO1 activity using the noncompetitive inhibitor lansoprazole (identified specifically as an inhibitor of PHOSPHO1^24^) during chick development induced ablation of mineralization in the lower limb bones.^23^ Together these data provided strong evidence that this novel phosphatase plays a critical role in the very first steps of skeletal biomineralization.

**Figure 1:**
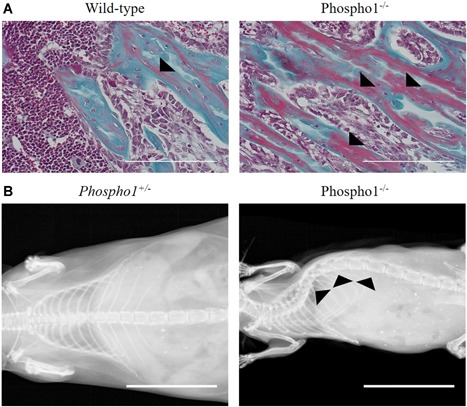
(*A*) Goldner's trichrome staining of wild‐type and *Phospho1* ^*–/–*^ tibial sections demonstrating osteoid accumulation in the trabeculae bone (red staining; black arrowheads). Tibias were dissected from 21‐day‐old C67BL/6 mice and fixed in 4% paraformaldehyde before decalcification in 10% EDTA in PBS and standard histological processing. Three‐micrometer (3‐µm) sections were cut using a rotary microtome and used for staining. Scale bars = 200 µm. (*B*) Radiographic images of 1‐year‐old *Phospho1* ^*+/–*^ and *Phospho1* ^*–/–*^ mice demonstrating thoracic scoliosis on ablation of *Phospho1* (black arrowheads). Whole‐body images were acquired using a MX20 Specimen Radiograph System (Faxitron Bioptics, LLC, Tucson, AZ, USA). Scale bars = 20 mm.

The generation of the PHOSPHO1 knockout mouse (*Phospho1* ^*–/–*^) enabled the detailed investigation of its phenotype and thereby allowed interrogation of the enzyme's specific function in the skeleton.^25^ Huesa and colleagues^26^ used a variety of compositional and biomechanical analyses to show that the mineral:matrix ratio of femora of juvenile *Phospho1* ^*–/–*^ mice was significantly lower than wild‐type controls, accompanied by plastic deformation upon three‐point bending and a reduced hardness and elastic modulus. A further investigation of the mechanical properties of these bones corroborated this high fracture toughness and also showed significantly higher indentation distance increases under reference point indentation compared to wild‐type controls.^27^ Histological examination of *Phospho1* ^*–/–*^ long bones revealed reduced mineralization in the trabecular compartment, whereas 10% to 15% of 10‐day‐old mice exhibited a complete lack of secondary ossification center development.^25^ These bones also demonstrated osteoid accumulation, a hallmark of hypophosphatasia (Fig. 1A). Interestingly, micro–computed tomography (µCT) revealed no differences between 4‐week‐old *Phospho1* ^*–/–*^ and wild‐type mice in bone volume relative to tissue volume (BV/TV), but rather a significantly reduced bone mineral density (BMD) that was accompanied by diverse spontaneous greenstick fractures and marked thoracic scoliosis^25^ (Fig. 1B). Similarly, PHOSPHO1 plays a critical role in fracture healing, with induced tibial fractures in *Phospho1* ^*–/–*^ mice demonstrating osteoid accumulation and elastic deformation upon loading at 4 weeks postsurgery.^28^

These findings have been extended to investigate the function of PHOSPHO1 in the development of bone's hydroxyapatite mineral phase at the smallest length scales. X‐ray diffraction (XRD) along with thermogravimetric analysis (TGA) revealed a lower bulk mineral content with significantly lower mineral:matrix ratio in *Phospho1* ^*–/–*^ femurs, also accompanied by a smaller apatite crystal size.^29^ Backscattered scanning electron microscopy (BSE‐SEM) furthermore revealed generalized hypomineralization relative to wild‐type mice in transverse tibial cross‐sections of the same animals.^29^ Further analysis of the *Phospho1* ^*–/–*^ microstructure exposed diffuse regions of hypomineralization in diaphyseal cortical and trabecular bone with large areas of osteoid accumulation.^30^ Interestingly, there was no discernible anatomical pattern between individual *Phospho1* ^*–/–*^ animals, but regions lacking mineral exhibited small focal areas of mineral nucleation at their borders, which failed to propagate more widely.

**Figure 2:**
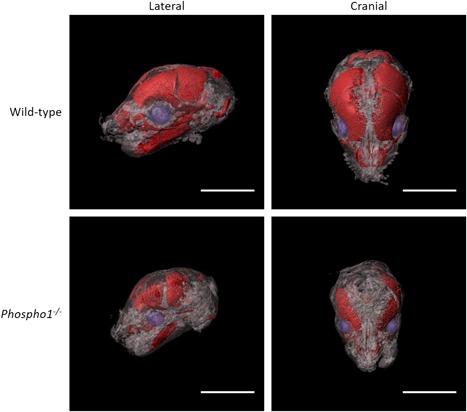
OPT reconstructions of mouse embryos at 17 days of gestation (E17) showing marked ablation of mineralization in bones of the skull in *Phospho1* ^*–/–*^ animals. E17 C67BL/6 wild‐type and *Phospho1* ^*–/–*^ embryos were culled using Schedule 1 methods, fixed in 4% paraformaldehyde and whole‐mount stained in a 0.001% Alizarin Red solution in 1% potassium hydroxide. Samples were embedded in 1% agarose and scanned using a Bioptonics 3001 OPT Scanner (Bioptonics, Medical Research Council Technology Group, UK). Data were reconstructed using NRecon (Bruker, Billerica, MA, USA) and visualized using Imaris (Bitplane, Belfast, UK). Scale bars = 10 mm. OPT = optical projection tomography.

In the very early studies of its function, characterization of the *Phospho1* ^*–/–*^ bone phenotype was performed in young juvenile mice, and therefore to examine the bone phenotype more fully Javaheri and colleagues^31^ investigated whether PHOSPHO1 plays a persistent long‐term role in the adult bone's biology where skeletal turnover is relatively slow. Using digital image correlation (DIC) the expected lower stiffness of *Phospho1* ^*–/–*^ was found, which was corrected with age. µCT analysis demonstrated several differences in tibial trabecular microarchitecture, including trabecular number and connectivity, between *Phospho1* ^*–/–*^ and wild‐type mice, which changed transiently across four age groups, with significant differences detected in the 5‐week and 34‐week age groups, but not in the 7‐week or 16‐week groups. Upon examination of the cortical bone, however, the authors noted a significant reduction in BV/TV in animals from 7 weeks of age that was not corrected over time. NanoCT scanning (0.6‐µm resolution) furthermore revealed a greater number of larger osteocyte lacunae, along with higher vascular porosity in *Phospho1* ^*–/–*^ tibias, a surprising finding which was compounded by an early increase in both *Pdpn* (embryonic day 11 [E11]) mRNA expression in primary osteoblast cell cultures, potentially indicating accelerated terminal osteocyte differentiation in these cells.^31^ PHOSPHO1 may therefore play a role in regulation of osteocytogenesis, which in turn may regulate bone microarchitecture in skeletally mature animals. Further research is required, however, to assess whether this relationship is mediated through reduced mineralization, or whether PHOSPHO1 may act within a distinct mechanism altogether. Our data also shows that the *Phospho1* ^*–/–*^ phenotype is present during skeletal development, with murine embryos exhibiting a reduced extent of mineralization across all bones during embryogenesis (unpublished data; Fig. 2).

One‐year‐old *Phospho1* ^*–/–*^ mice showed reduced plasma concentrations of TNAP with a concomitant increase in ectonucleotide pyrophosphatase/phosphodiesterase1 (NPP1), thereby resulting in significantly higher PP_i_ concentrations compared with wild‐type controls.^25^ Intriguingly, high serum PP_i_ is a key characteristic of infantile hypophosphatasia in humans, with the resulting inhibition of mineralization attributed to the ensuing rickets/osteomalacia.^17^, ^32^ Yadav and colleagues^25^ attempted to rescue the *Phospho1* ^*–/–*^ phenotype by cross‐breeding with mice overexpressing TNAP (*ApoE‐Tnap*) and thereby reduce PP_i_ concentration. Although the authors did indeed observe an approximately fourfold increase in TNAP in plasma and a significant reduction in PP_i_, the hypomineralized *Phospho1* ^*–/–*^ phenotype was not corrected, with animals exhibiting persistent skeletal defects at 7 months. Furthermore PHOSHO1; TNAP double knockout (*Phospho1* ^*–/–*^; *Alpl* ^*–/–*^) mice exhibited complete ablation of skeletal mineralization and perinatal lethality.^25^ These data were augmented and confirmed in vitro with osteoblast‐like MC3T3‐E1 cell lines (clones 14 and 24) along with ex vivo metatarsal cultures using specific PHOSPHO1 and TNAP inhibitors in culture.^33^

PP_i_ is known to regulate the expression of other mineralization‐associated proteins, particularly osteopontin (OPN; *Spp1*).^34^, ^35^, ^36^ Along with PP_i_, OPN is another potent mineralization inhibitor.^37^, ^38^, ^39^ The protein's inhibitory effects are mediated through its phosphorylation status,^40^, ^41^ and this has been shown to be regulated by TNAP.^42^ Significantly elevated OPN, which also exhibited a greater degree of phosphorylation compared to controls, was found at the protein and gene levels in serum and spinal lysates of *Phospho1* ^*–/–*^ mice at 1 months and 3 months postnatally.^43^ Interestingly, no differences were observed in femoral lysates. Yadav and colleagues^43^ investigated the interplay between PHOSPHO1 and OPN through the generation of *Phospho1* ^*–/–*^; *Spp1* ^*–/–*^ mice and found a partial rescue of the *Phospho1* ^*–/–*^ phenotype, with animals at 1 month and 3 months of age exhibiting a reduction in the typical hyperosteoidosis and thoracic scoliosis that characterizes the single knockout animal. The *Phospho1* ^*–/–*^ hypomineralized mouse phenotype is therefore partially attributable to an increased expression of OPN, which may obstruct mineralization during bone formation. This is likely exacerbated by a relative hyperphosphorylation of OPN, mediated by the established reduced expression of *Alpl* in *Phospho1* ^*–/–*^ mice.^25^, ^43^

Collectively these findings establish a nonredundant role of PHOSPHO1 in mediating biomineralization of the skeleton, as well as its regulated synergy with TNAP, along with other mineralization‐associated factors including PP_i_ and OPN, as part of this process.

## Dental Mineralization

Along with its established role in bone biomineralization, PHOSPHO1 has also been implicated in the mineralization process of the dentition. McKee and colleagues^44^ studied dentin formation in perinatal mice and found localization of PHOSPHO1 to odontoblasts during the very first steps of dentin formation. *Phospho1* ^*–/–*^ mice exhibited reduced mineralization in the dentin by histology, µCT, and radiography, a phenotype that was aggravated by the additional deletion of one *Alpl* allele (*Phospho1* ^*–/–*^; *Alpl* ^*+/–*^). Others have examined PHOSPHO1 function in the other dental hard tissues, namely cementum and enamel. PHOSPHO1 was found to show expression in osteoblasts and cementoblasts of alveolar bone and cementum, respectively, during their mineralization phases where osteoid accumulation was evident in the bone and was accompanied by delayed mineralization of the cellular cementum in *Phospho1* ^*–/–*^ animals.^45^ Intriguingly, acellular cementum formation was normal, despite downregulation of *Phospho1* ^*–/–*^ in a bone sialoprotein knockout model in this region.^46^ PHOSPHO1 is therefore an enzyme critical for the integrity of the periodontal tissue interface, along with normal development of these hard tissues. In enamel, *Phospho1* ^*–/–*^ mice exhibited a 25% increase in tissue volume, with a significantly reduced level of mineralization.^47^ The authors furthermore reported that ablation of PHOSPHO1 caused loss of enamel prism morphology and an impaired crystal organization compared to wild‐type mice.

At the level of local cellular regulation, control of mineralization through the PHOSPHO1 pathway has been shown to be influenced by the TRPS1 transcription factor in an odontoblast‐like cell line.^48^ Mutations in the *Trps1* gene are associated with trichorhinophalangeal syndrome in humans and manifest craniofacial and skeletal dysplasias associated with defective endochondral mineralization.^49^, ^50^, ^51^ Loss of TRPS1 has also been implicated in abnormal tooth development and mineralization.^52^ Kuzynski and colleagues^48^ overexpressed *Trps1* in preodontoblastic 17IIA11 cells and found reduced mineralization compared with controls. Interestingly, reduction of *Trps1* expression also ablated mineralization and was associated with downregulation of *Phospho1* and *Alpl* expression. The authors proposed that TRPS1 acts to repress mineralization associated genes in dentin in a biphasic manner to first inhibit ectopic mineralization, and further to prevent hypermineralization by modulation of osteogenic gene expression, including *Phospho1*.

## The PHOSPHO1 Mineralization Mechanism: Matrix Vesicle–Mediated Biomineralization

Matrix vesicles (MVs) are membrane‐bound nanospherical bodies of approximately 100 to 300 nm in diameter that are typically rich in lipids and proteins known to chelate Ca^2+^ and P_i_, and which are associated with both physiological and pathological biomineralization.^53^ Their function continues to be remain controversial since their discovery in 1967 in growth plate cartilage,^54^, ^55^ with some authors attributing these as specimen‐preparation artifacts.^56^ Nevertheless, many in vitro and in vivo studies have shown the first mineral crystals in diverse mineralized tissues such as bone, dentin, cartilage, and mineralized vasculature are associated with these structures in the extracellular matrix (ECM).^55^, ^57^, ^58^, ^59^, ^60^, ^61^, ^62^, ^63^

The structure and function of MVs in skeletal and vascular mineralization has recently been reviewed by several authors.^53^, ^64^, ^65^, ^66^, ^67^ The biogenesis of these vesicles may occur through multiple proposed mechanisms; however, the most prevalent theories include polarized budding from the parental cell membrane, or from microvilli on the cell surface, as demonstrated in hypertrophic chondrocytes and SaOS‐2 osteoblast‐like cells.^68^, ^69^, ^70^, ^71^ The theorized function of MVs in biomineralization is to facilitate a localized concentration of Ca^2+^ and P_i_, protected from the ECM, from which hydroxyapatite or its precursors may form.^53^ Vesicles may also assist in concentrating P_i_ to engender a P_i_:PP_i_ ratio that is permissible for mineralization.^72^ MVs are known to be enriched in Ca^2+^ by way of their intracellular biogenesis from mitochondria under oxidative stress, at least in growth‐plate chondrocytes.^73^, ^74^ Recently published research by Chaudhary and colleagues^75^ may correlate well with these findings, showing that P_i_ stimulates release of MVs from osteogenic cells, although the authors did not comment on their mitochondrial origin.

**Figure 3:**
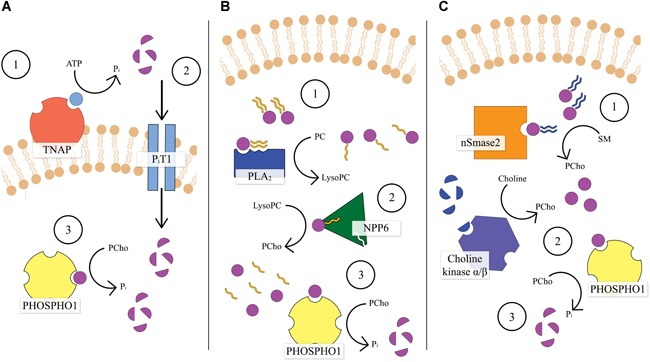
Schematic diagram illustrating the hypothesized mechanism of PHOSPHO1 function within MVs. (*A*) PHOSPHO1 functions synergistically with TNAP: (1) TNAP hydrolyses its substrates to produce P_i_ extravesicularly; (2) extravesicular P_i_ is transported into the MV via P_i_T1; (3) PHOSPHO1 hydrolyses Pcho intravesicularly to further accumulate P_i_. (*B*) Generation of PHOSPHO1 substrates within MVs: (1) an unidentified PLA_2_ converts PC from the vesicle membrane to lysoPC; (2) NPP6 subsequently catalyses the hydrolysis of lysoPC to generate Pcho; (3) PHOSPHO1 liberates P_i_ from PCho. (*C*) Alternative pathways of PCho generation: (1) nSmase2 breaks down SM from the MV membrane to form Pcho; (2) the α/β choline kinases phosphorylate choline to form PCho; (3) PHOSPHO1 generates P_i_ from PCho.

P_i_ accumulation within MVs is thought to be mediated by extravesicular and intravesicular phosphatases, of which PHOSPHO1 plays a critical role.^11^, ^20^, ^25^, ^26^, ^76^, ^77^, ^78^ Stewart and colleagues^77^ were the first to suggest PHOSPHO1 as a major intravesicular phosphatase, showing its presence in MVs isolated from embryonic chick growth plates. This was more recently confirmed in MVs isolated from the ECM of both cultured MC3T3 and SaOS‐2 osteoblast‐like cells using Western blotting^75^ and quantitative proteomics.^71^, ^79^ Further work using both growth plate–derived and primary osteoblast**–**derived vesicles established that although TNAP does indeed liberate P_i_ extravesicularly, PHOSPHO1 is biochemically active intravesicularly.^24^, ^80^ Therefore, it is hypothesized that accumulation of P_i_ inside MVs occurs via a combination of the intravesicular action of PHOSPHO1 and intravesicular trafficking of TNAP‐generated P_i_ via a Type III Na‐P_i_ co‐transporter, P_i_T1 (encoded by the *Slc20a1* gene in mice)^77^, ^80^ (Fig. 3A). Experimental evidence for this hypothesis comes from the work of Yadav and colleagues,^81^ who generated a cartilage specific P_i_T1 knockout mouse driven by a *col2a1*‐Cre on a *Phospho1*‐null background (*Phospho1* ^*–/–*^; *P*_*i*_ *T1* ^*Col2/Col2*^). The authors report an exacerbation of the *Phospho1* ^*–/–*^ phenotype, including growth plate defects, extensive hyperosteoidosis, decreased BMD, and impaired mechanical properties. MVs isolated from differentiating chondrocytes in these animals furthermore demonstrated a loss of their capacity to nucleate hydroxyapatite crystals compared to both *Phospho1* ^*–/–*^ and wild‐type controls.^81^ Interestingly, both *Phospho1* ^*–/–*^ and *Phospho1* ^*–/–*^; *P*_*i*_ *T1* ^*Col2/Col2*^ MV were reduced in numbers compared to wild‐types, potentially indicating a role for PHOSPHO1 in MV biogenesis. Together, these studies, along with those characterizing the *Alpl* ^*–/–*^ and *Phospho1* ^*–/–*^; *Alpl* ^*–/–*^ mice,^16^, ^17^, ^20^, ^21^, ^25^ afford good evidence for this mechanism integrating the PHOSPHO1 and TNAP P_i_ generation pathways.

Although this work provides a strong evidence base for the pivotal role of PHOSPHO1 in vesicle‐mediated biomineralization, the specific biochemical pathway within it by which it achieves intravesicular P_i_ liberation remains unclear. Stewart and colleagues^82^ proposed a mechanism through which PHOSPHO1's substrates PEA and PCho may be generated intravesicularly by enzymatic action upon the vesicle's phospholipid membrane, as mediated by a phospholipase A_2_ (PLA_2_) and ectonucleotide pyrophosphatase/phosphodiesterase 6 (NPP6)^82^ (Fig. 3B). The PLA_2_ family of enzymes catalyze cleavage of the acyl group at the *sn*‐2 acyl position of glycerophospholipids resulting in a free fatty acid and lysophospholipid (LPL).^83^, ^84^, ^85^ These enzymes may therefore act to breakdown phosphatidylcholine (PC) and phosphatidylethanolamine (PE) in the MV membrane, forming lysophosphatidylethanolamine and lysophosphocholine LPLs, respectively, along with arachidonic acid.^82^ Indeed, the MV membrane has been shown to be enriched in phospholipids containing PCho and PEA, which progressively decline during mineralization,^74^, ^86^, ^87^ whereas PCho was also identified as an abundant metabolite in developing mouse long bones by matrix‐assisted laser desorption/ionization‐imaging mass spectrometry.^88^ There are upward of 30 identified mammalian PLA_2_ enzymes that exhibit a huge range of localizations (including secreted, cytosolic, and lysosomal groups) and have been shown to be involved with many physiological and pathological processes.^83^, ^84^, ^85^ Mebarek and colleagues^89^ comprehensively reviewed the evidence for the role of phospholipases in mineralization, noting several experimental studies confirming expression of both secreted and cytosolic PLA_2_s in chondrocytes and osteoblasts where they play several roles. Although some specific PLA_2_s have been shown to have an effect on bone formation,^90^ it is currently unclear whether these act directly within the mineralization process. Therefore, further research is required to identify specific candidate proteins that fulfill this niche.

A second enzymatic processing phase is hypothesized to convert generated LPL to PCho for direct hydrolysis by PHOSPHO1, mediated by NPP6.^82^ NPP6 is a member of the nucleotide pyrophosphatase/phosphodiesterase family and has been shown to possess lysophospholipase C activity, catalyzing the conversion of lysophosphocholine with a monoacylglycerol by‐product.^91^, ^92^, ^93^ Expression of NPP6 has been demonstrated in bone tissue lysate and was immunolocalized to hypertrophic chondrocytes and forming bone surfaces.^82^ Specific localization of NPP6 to MVs has yet to be established, however.

Alternative biochemical pathways through which PHOSPHO1 substrates may be generated have also been proposed (Fig. 3C). Neutral sphingomyelinase 2 (nSmase2) is encoded by the *Smpd3* gene in mice and is capable of catalyzing the hydrolysis of another MV membrane phospholipid, sphingomyelin, to produce PCho with a ceramide byproduct.^94^ The *fro* mutation in the *Smpd3* gene generates a distinctive phenotype with extensive musculoskeletal defects resulting in dwarfism.^95^, ^96^ Khavandgar and colleagues^97^ demonstrated that the *fro/fro* mouse exhibits delayed mineralization of the long bones and calvaria, along with impaired hypertrophy in growth plate chondrocytes during embryonic development. Using tissue‐specific mouse knockout models it was also established that *Smpd3* expression is required in both osteoblasts and chondrocytes in a cell‐autonomous manner for normal bone development.^97^, ^98^ These effects were moreover shown to influence mineralization during tooth development and fracture healing.^99^, ^100^ Like PC and PE, sphingomyelin is enriched in MV preparations and declines during mineralization,^74^, ^86^, ^87^ whereas the nSmase2 enzyme has been localized to MV isolates in conjunction with PHOSPHO1.^79^ nSmase2‐mediated production of PCho may therefore provide another pathway for the intravesicular generation of PHOSPHO1 substrates. Another potential alternative pathway includes generation of PCho by phosphorylation of choline through the action of the α and β choline kinases (encoded by the *Chkα* and *Chkβ* genes, respectively). Although mice lacking *Chkα* display lethality during embryonic development,^101^ the *Chkβ* ^*–/–*^ mouse exhibits forelimb deformities and delayed mineralization, accompanied by an extended and disorganized hypertrophic zone within the growth plates at the distal radius and ulna.^102^ PCho was also shown to be reduced by ∼75% in primary chondrocytes isolated from these animals.^102^ Intriguingly, PHOSPHO1 was upregulated in *Chkβ* ^*–/–*^ primary chondrocytes, potentially indicating compensation for a restricted substrate availability.^102^ Both *Chkα* and *Chkβ* were found to be expressed in human osteoblast‐like MG‐63 cells, and gene silencing of *Chkα* resulted in a reduction of ∼70% in cellular PCho with an accompanied inhibition of TNAP activity and mineralization in culture.^103^

## Endocrinology and Regulation

As a critical effector of biomineralization it is likely that PHOSPHO1 expression and function will be stringently controlled at both the local and systemic level. As a major organ, and one which is metabolically expensive to produce and maintain, it is well established that bone formation and resorption are tightly controlled by many factors during development and in adulthood, some of the most significant of which include the sex steroids,^104^, ^105^, ^106^ vitamin D,^107^, ^108^, ^109^ and parathyroid hormone (PTH).^110^, ^111^, ^112^ Whether the observed differences in mineralization caused by regulatory influences are caused wholly or partly by control of PHOSPHO1 or other associated proteins is as yet unknown.

Some evidence for the systemic regulation of PHOSPHO1 comes from work surrounding the effect of PTH on osteoblasts, with RNA‐seq in an osteocyte‐like cell line (IDG‐SW3 cells) stimulated with PTH in culture demonstrated an effect on both *Phospho1* and *Smpd3* expression.^113^ Houston and colleagues^114^ extended these analyses, examining the expression of *Phospho1*, *Smpd3*, and *Alpl* specifically in MC3T3 osteoblast‐like cells and ex vivo calvaria culture models with continuous PTH exposure. The authors report rapid and coordinate downregulation of *Phospho1* and *Smpd3* expression after the addition of PTH to culture media, which was likely regulated through the cAMP/PKA signaling pathway. This downregulation of both *Phospho1* and *Smpd3* was independently found in Kusa 4b10 cells and in young rats exposed to PTH and PTH‐related protein 1 (PTHrP).^115^, ^116^ The catabolic effects of continuous PTH on the skeleton are well demonstrated in human conditions such as hyperparathyroidism; however, much research has associated this with the upregulation of osteoclastogenesis through the RANKL/OPG axis.^117^, ^118^, ^119^ These data may indicate, however, a simultaneous mechanism effecting inhibition of bone formation as mediated by PHOSPHO1. The transcription and posttranslational modification (phosphorylation) of Runx2, the transcription factor and master regulator of osteoblast differentiation, is also strongly enhanced by PTH.^120^ Interestingly, the overexpression of *Runx2* in mouse limb bud cultures simultaneously enhances the expression of both *Phospho1* and *Smpd3*.^121^

Collectively, these data surrounding PTH tentatively suggest that control of bone biomineralization may occur by modulating PHOSPHO1 expression, and thereby the MV‐mediated biomineralization mechanism. This relationship is, however, currently far from explicit and much further research is required to establish the regulatory mechanisms behind PHOSPHO1 expression and function within the context of the MV.

## PHOSPHO1 in Pathologies of Mineralization

Aside from its central function in mediating physiological biomineralization, PHOSPHO1 may also have a role to play in the onset of pathological mineral formation; for example in vascular calcification. *Phospho1* is upregulated during mineralization of vascular smooth muscle cells (VMSCs) in culture, whereas *Phospho1* ^*–/–*^ VSMCs exhibited a significant reduction in mineral generation.^122^ Also, the PHOSPHO1 inhibitor, MLS‐0263839 reduced calcification of cultured VSMCs by ∼60% and when combined with the TNAP inhibitor MLS‐0038949, calcification of VSMCs was reduced by ∼80%.^122^ *Phospho1* is also upregulated during atherosclerotic vascular calcification in a rabbit model.^123^ Furthermore, Hortells and colleagues^124^ induced aortic calcification in a rat nephrectomy model with a 1.2% phosphorous diet for 12 weeks postsurgery and observed significant upregulation of *Phospho1* expression in animals exhibiting calcification, along with several other mineralization‐associated genes. These studies suggest that PHOSPHO1 has a critical role in VSMC mineralization and that “phosphatase inhibition” may offer therapeutic strategies to mitigate vascular calcification.

Several authors have investigated the process responsible for vascular calcification, with some implicating PHOSPHO1 and TNAP as part of the MV mechanism.^125^, ^126^ Scanning electron microscopy (SEM) and focused ion beam scanning electron microscopy (FIB‐SEM) of human calcified valves and vasculature revealed nanospherical particles composed of crystalline calcium phosphate.^127^ Using in vitro models together with calcified human tissue, these particles were later implicated in the formation of mineralized atherosclerotic plaques through their initial aggregation and nucleation of the mineral phase.^128^ Furthermore, particles isolated from mineralized aortas were subsequently shown to induce pathological changes in valvular endothelial cells (VECs) and valvular interstitial cells (VICs) in culture.^129^ These studies demonstrate compelling similarities between calcified particles found in the vasculature and MVs. The localization of PHOSPHO1 or other MV‐associated enzymes with these structures has not as yet, however, been confirmed and so the mechanism behind their generation remains elusive.

There is now substantial evidence that the mineralization status of the subchondral bone is altered in osteoarthritis, and that this may lead to modified mechanical integrity, engendering greater articular cartilage degeneration.^130^ *Phospho1* is upregulated during chondrocyte differentiation,^131^ and aged hypomineralized *Phospho1* ^*–/–*^ mice exhibit increased articular cartilage degradation and osteophyte formation, when compared to the age‐matched wild‐type mice.^132^ Further, noninvasive loading of *Phospho1* ^*–/–*^ mouse knee joints revealed diminished loading‐induced changes in the subchondral bone plate thickness and epiphyseal trabecular bone microarchitecture.^132^ Together, these data suggest that the hypomineralized bone phenotype in *Phospho1* ^*–/–*^ mice provokes osteoarthritis pathology. This therefore implies that local modifications in the bone matrix mineralization may underpin subchondral bone sclerosis in osteoarthritis; however, further analyses are required to fully define this relationship.

There are also more speculative roles for PHOSPHO1 in osteoarthritis because of its role as a phosphatase capable of hydrolyzing PCho and PEA. Lipidomic analysis of the synovial fluid from patients with osteoarthritis suggests that alterations in the phospholipid composition and concentrations are associated with disease development, due to the lubricating function of the synovial fluid in the joint.^133^ Specifically, PC concentrations are increased in the synovial fluid of both early (2.7‐fold) and late (5.4‐fold) osteoarthritic patients in comparison to controls.^133^ Similarly, there has been reported high activity of PLA_2_, thought to break down PC, in the synovial fluid of osteoarthritic patients^134^ and a role for PLA_2_, in articular cartilage chondrocyte function.^135^ It has been suggested that the ratio of PC to lysophosphatidylcholine is a good diagnostic marker for rheumatoid arthritis.^136^ However, whether this is the case for osteoarthritis and the potential involvement of PHOSPHO1 in this is yet to be established.

## Other Physiological Roles

In the 20 years since its characterization, the vast majority of research focusing on PHOSPHO1 has concentrated on biomineralization. However, as a phosphatase capable of hydrolyzing PCho and PEA with the generation of metabolites such as choline, PHOSPHO1 has the potential for activity in many other body systems. PC and PE are two of the most abundant lipids in the body, comprising 40% to 50% and 15% to 20%, respectively, of total cellular phospholipids of any given tissue in mammals.^137^ These molecules are therefore of critical importance in a huge variety of biological systems, from the integrity of plasma membranes, and other structures such as vesicles, lipoproteins and chylomicrons, to regulating the activity of proteins at the cell membrane.^137^, ^138^ Interestingly, the breakdown products from PC in particular have also been implicated as signaling molecules.^139^, ^140^ Although speculative, it is therefore an intriguing possibility that PHOSPHO1 may have a role to play in the biosynthesis and catalysis pathways of these phospholipids, regulating substrate availability. Choline is the breakdown product from PHOSPHO1 activity on the LPL lysophosphatidylcholine derived from PC, and is an essential dietary nutrient in mammals. Although choline is primarily used in PC biosynthesis, it may also be used to generate betaine in the liver and kidney, or acetylated to form the neurotransmitter acetylcholine in the brain.^141^ PHOSPHO1 may therefore be an appealing candidate to act within a secondary mechanism of choline homeostasis in these and other tissues. Indeed, gut enteroids maintained in choline‐deficient media exhibited hypomethylation at 3′ CpG islands within the *Phospho1* gene, potentially regulating gene expression.^142^

One system within which PHOSPHO1 plays a significant role in this regard is erythropoiesis. An expression quantitative trait locus (eQTL) analysis initially found that single‐nucleotide polymorphisms (SNPs) associated with β‐thalassemia in human peripheral blood samples were also associated with changes in *PHOSPHO1* expression.^143^ PHOSPHO1 was also found to be substantially enriched in erythroblasts undergoing differentiation.^144^ Subsequently Huang and colleagues^145^ investigated PHOSPHO1 in terminal erythropoiesis; a process during which the phospholipid composition of red cells is substantially altered. *PHOSPHO1* expression was significantly upregulated during erythroblast differentiation, and moreover loss of PHOSPHO1 induced defective erythropoiesis accompanied by a lack of choline generation and an increased phosphocholine:choline ratio.

Several other studies have found intriguing associations between *PHOSPHO1* expression and disorders of altered energy metabolism such as diabetes and obesity. Epigenome‐wide association studies (EWAS) have found significant associations between methylation at loci within the *PHOSPHO1* and the future risk of type‐2 diabetes in human cohorts.^146^, ^147^ Indeed, Willmer and colleagues^148^ highlighted differential methylation at sites within the *PHOSPHO1* gene as a potentially useful biomarker for clinical application in the early detection of type‐2 diabetes. Moreover, increased methylation was positively correlated with high density lipoprotein (HDL) concentration in blood and was decreased in muscle tissue from diabetic patients.^147^ This relationship with HDL concentration was also reproduced independently in an EWAS focused on serum lipid profiles conducted in a separate cohort.^149^ Relatedly, Wu and colleagues^150^ further found an association between *PHOSPHO1* and SNPs relating to clinical measures of obesity in a Chinese population. These studies provide a promising avenue of investigation for future research to consider the mechanisms through which PHOSPHO1 may act to influence lipid metabolism and disorders of its dysregulation such as diabetes and obesity. Supporting this idea, changes in *Phospho1* expression have been found in several studies examining the thermogenic brown adipose tissue (BAT) and the browning of white adipose tissue (WAT), of great relevance to obesity. *Phospho1* is expressed at a higher level in BAT than WAT,^151^ and suppression of fatty acid oxidation in adipose tissues causes a downregulation in expression during cold challenge.^152^ Furthermore, studies that have investigated creatine metabolism as an alternative pathway for thermogenesis in beige fat have implicated PHOSPHO1 in this process, although the specific mechanism remains elusive.^153^, ^154^

## Inhibition of PHOSPHO1—a Therapeutic Target?

The LOPAC and Spectrum chemical reference libraries were used to identify potential chemical inhibitors of PHOSPHO1 and semiautomated high‐throughput chemical screening used to test inhibition of PEA hydrolysis.^24^ Of those identified, three molecules, Lansoprazole, Ebseleln, and SCH202676, exhibited noncompetitive inhibition of recombinant PHOSPHO1 activity by 80% or more.^24^ This study showed that neither Lansoprazole nor SCH202676 inhibited TNAP activity, but Lansoprazole has recently been shown to act as a noncompetitive inhibitor of TNAP.^155^ Following this, Bravo and colleagues^156^ synthesized a series of benzoisothiazolone inhibitors of PHOSPHO1; the final selection from these inhibitors was tested and passed medicinal chemistry criteria in deliverability, metabolic stability, solubility, and permeability, while also demonstrating no cellular toxicity.

The development of these compounds opens the potential for inhibition of PHOSPHO1 activity as a therapeutic intervention. Although there has been no experimental research exploring this possibility to date, the imputation of PHOSPHO1's association with pathologies in both biomineralization and lipid metabolism may make it an attractive drug target in the future. In terms of disorders of pathological mineralization, although the PHOSPHO1‐mediated mineralization mechanism is critical for the proper development of the skeleton in immature organisms, as has been discussed here, there is currently no evidence to suggest that it plays an active role in maintenance of the skeleton in adulthood. Ablation of mineralization in the adult may be desirable when attempting to control soft tissue mineralization, eg, vascular calcification and also ectopic bone formation, as is the case in several musculoskeletal disorders including osteoarthritis. In these instances pharmacological inhibition of PHOSPHO1 may prove to represent an effective intervention that also entails limited adverse off‐target effects.

## Outlook

Over the past 20 years PHOSPHO1 has been an increasing focus of research in the bone biology and biomineralization communities. Work performed in our and in other groups has succeeded in establishing its canonical role in MV‐mediated biomineralization of the skeleton and dentition, while opening new fields of investigation into its function in other body systems. Many questions remain to be answered surrounding aspects of PHOSPHO1's biology, including detailed elucidation of its upstream biochemical mechanism, targeting of the enzyme to MVs, how local and systemic regulation is integrated with other aspects of bone biology during the bone formation process, and the precise role of PHOSPHO1in musculoskeletal disease. Future studies integrating aspects of structural biology and biomineralization with cellular and molecular biology and endocrinology will give us a holistic appreciation of these processes and contribute to our understanding in this fundamental aspect of bone biology.

## Disclosures

All authors state that they have no conflicts of interest.

## References

[1] Suchacki K , Roberts F , Lovdel A , et al. Skeletal energy homeostasis: a paradigm of endocrine discovery. J Endocrinol. 2017;234(1):R67–79. 28455432 10.1530/JOE-17-0147

[2] Reznikov N , Shahar R , Weiner S. Bone hierarchical structure in three dimensions. Acta Biomater. 2014;10(9):3815–26. 24914825 10.1016/j.actbio.2014.05.024

[3] Reznikov N , Shahar R , Weiner S . Three‐dimensional structure of human lamellar bone: the presence of two different materials and new insights into the hierarchical organization. Bone. 2014;59:93–104. 24211799 10.1016/j.bone.2013.10.023

[4] Houston B , Seawright E , Jefferies D , et al. Identification and cloning of a novel phosphatase expressed at high levels in differentiating growth plate chondrocytes. Biochim Biophys Acta Mol Cell Res. 1999;1448(3):500–6. 10.1016/s0167-4889(98)00153-0 9990301

[5] Houston B , Paton I , Burt D , Farquharson C . Chromosomal localization of the chicken and mammalian orthologues of the orphan phosphatase PHOSPHO1 gene. Anim Genet. 2002;33(6):451–4. 12464021 10.1046/j.1365-2052.2002.00900.x

[6] Stewart AJ , Schmid R , Blindauer CA , Paisey SJ , Farquharson C . Comparative modelling of human PHOSPHO1 reveals a new group of phosphatases within the haloacid dehalogenase superfamily. Protein Eng. 2003;16(12):889–95. 14983068 10.1093/protein/gzg126

[7] Baldwin JC , Karthikeyan AS , Raghothama KG . LEPS2 a phosphorus starvation‐induced novel acid phosphatase from tomato. Plant Physiol. 2001;125(2):728–37. 11161030 10.1104/pp.125.2.728 PMC64874

[8] Stenzel I , Ziethe K , Schurath J , Hertel SC , Bosse D , Köck M . Differential expression of the LePS2 phosphatase gene family in response to phosphate availability, pathogen infection and during development. Physiol Plant. 2003;118(1):138–46. 12702022 10.1034/j.1399-3054.2003.00091.x

[9] Baldwin JC , Karthikeyan AS , Cao A , Raghothama KG . Biochemical and molecular analysis of LePS2; 1: a phosphate starvation induced protein phosphatase gene from tomato. Planta. 2008;228(2):273. 18458947 10.1007/s00425-008-0736-y

[10] Wang W , Kim R , Jancarik J , Yokota H , Kim S‐H . Crystal structure of phosphoserine phosphatase from *Methanococcus jannaschii*, a hyperthermophile, at 1.8 Å resolution. Structure. 2001;9(1):65–71. 11342136 10.1016/s0969-2126(00)00558-x

[11] Roberts SJ , Stewart AJ , Sadler PJ , Farquharson C . Human PHOSPHO1 exhibits high specific phosphoethanolamine and phosphocholine phosphatase activities. Biochem J. 2004;382(1):59–65. 15175005 10.1042/BJ20040511 PMC1133915

[12] Roberts SJ , Stewart AJ , Schmid R , et al. Probing the substrate specificities of human PHOSPHO1 and PHOSPHO2. Biochim Biophys Acta Proteins Proteom. 2005;1752(1):73–82. 10.1016/j.bbapap.2005.06.009 16054448

[13] Nielson CM , Zmuda JM , Carlos AS , Wagoner WJ , Larson EA , Orwoll ES , et al. Rare coding variants in ALPL are associated with low serum alkaline phosphatase and low bone mineral density. J Bone Miner Res. 2012;27(1):93–103. 21956185 10.1002/jbmr.527 PMC3810303

[14] Ozono K , Michigami T . Hypophosphatasia now draws more attention of both clinicians and researchers: a Commentary on prevelance of c. 1559delT in ALPL, a common mutation resulting in the perinatal (lethal) form of hypophosphatasias in Japanese and effects of the mutation on heterozygous carriers. J Hum Genet. 2011;56(3):174. 21307860 10.1038/jhg.2011.6

[15] Ermakov S , Toliat MR , Cohen Z , et al. Association of ALPL and ENPP1 gene polymorphisms with bone strength related skeletal traits in a Chuvashian population. Bone. 2010;46(5):1244–50. 19931660 10.1016/j.bone.2009.11.018

[16] Narisawa S , Fröhlander N , Millán JL . Inactivation of two mouse alkaline phosphatase genes and establishment of a model of infantile hypophosphatasia. Dev Dyn. 1997;208(3):432–46. 9056646 10.1002/(SICI)1097-0177(199703)208:3<432::AID-AJA13>3.0.CO;2-1

[17] Fedde KN , Blair L , Silverstein J , et al. Alkaline phosphatase knock‐out mice recapitulate the metabolic and skeletal defects of infantile hypophosphatasia. J Bone Miner Res. 1999;14(12):2015–26. 10620060 10.1359/jbmr.1999.14.12.2015 PMC3049802

[18] Liu J , Nam HK , Campbell C , da Silva Gasque KC , Millán JL , Hatch NE . Tissue‐nonspecific alkaline phosphatase deficiency causes abnormal craniofacial bone development in the Alpl−/− mouse model of infantile hypophosphatasia. Bone. 2014;67:81–94. 25014884 10.1016/j.bone.2014.06.040 PMC4149826

[19] Foster B , Nagatomo K , Tso H , Tran A , Nociti F , Narisawa S , et al. Tooth root dentin mineralization defects in a mouse model of hypophosphatasia. J Bone Miner Res. 2013;28(2):271–82. 22991301 10.1002/jbmr.1767 PMC3541444

[20] Anderson HC , Sipe JB , Hessle L , et al. Impaired calcification around matrix vesicles of growth plate and bone in alkaline phosphatase‐deficient mice. Am J Pathol. 2004;164(3):841–7. 14982838 10.1016/s0002-9440(10)63172-0 PMC1613274

[21] Anderson HC , Hsu HH , Morris DC , Fedde KN , Whyte MP . Matrix vesicles in osteomalacic hypophosphatasia bone contain apatite‐like mineral crystals. Am J Pathol. 1997;151(6):1555. 9403706 PMC1858375

[22] Houston B , Stewart AJ , Farquharson C . PHOSPHO1—a novel phosphatase specifically expressed at sites of mineralisation in bone and cartilage. Bone. 2004;34(4):629–37. 15050893 10.1016/j.bone.2003.12.023

[23] MacRae VE , Davey MG , McTeir L , et al. Inhibition of PHOSPHO1 activity results in impaired skeletal mineralization during limb development of the chick. Bone. 2010;46(4):1146–55. 20053388 10.1016/j.bone.2009.12.018 PMC2842458

[24] Roberts S , Narisawa S , Harmey D , Millán JL , Farquharson C . Functional involvement of PHOSPHO1 in matrix vesicle–mediated skeletal mineralization. J Bone Miner Res. 2007;22(4):617–27. 17227223 10.1359/jbmr.070108

[25] Yadav MC , Simao AMS , Narisawa S , et al. Loss of skeletal mineralization by the simultaneous ablation of PHOSPHO1 and alkaline phosphatase function: a unified model of the mechanisms of initiation of skeletal calcification. J Bone Miner Res. 2011;26(2):286–97. 20684022 10.1002/jbmr.195 PMC3179344

[26] Huesa C , Yadav MC , Finnilä MA , et al. PHOSPHO1 is essential for mechanically competent mineralization and the avoidance of spontaneous fractures. Bone. 2011;48(5):1066–74. 21272676 10.1016/j.bone.2011.01.010 PMC3078982

[27] Carriero A , Bruse JL , Oldknow KJ , Millán JL , Farquharson C , Shefelbine SJ . Reference point indentation is not indicative of whole mouse bone measures of stress intensity fracture toughness. Bone. 2014;69:174–9. 25280470 10.1016/j.bone.2014.09.020 PMC4228060

[28] Morcos M , Al‐Jallad H , Li J , et al. PHOSPHO1 is essential for normal bone fracture healing: an animal study. Bone Joint Res. 2018;7(6):397–405. 30034793 10.1302/2046-3758.76.BJR-2017-0140.R2 PMC6035360

[29] Rodriguez‐Florez N , Garcia‐Tunon E , Mukadam Q , et al. An investigation of the mineral in ductile and brittle cortical mouse bone. J Bone Miner Res. 2015;30(5):786–95. 25418329 10.1002/jbmr.2414 PMC4507744

[30] Boyde A , Staines KA , Javaheri B , Millan JL , Pitsillides AA , Farquharson C . A distinctive patchy osteomalacia characterises Phospho1‐deficient mice. J Anat. 2017;231(2):298–308. 28737011 10.1111/joa.12628 PMC5522900

[31] Javaheri B , Carriero A , Staines K , et al. Phospho1 deficiency transiently modifies bone architecture yet produces consistent modification in osteocyte differentiation and vascular porosity with ageing. Bone. 2015;81:277–91. 26232374 10.1016/j.bone.2015.07.035 PMC4652607

[32] Whyte MP . Hypophosphatasia and the role of alkaline phosphatase in skeletal mineralization. Endocr Rev. 1994;15(4):439–61. 7988481 10.1210/edrv-15-4-439

[33] Huesa C , Houston D , Kiffer‐Moreira T , Yadav MC , Millan JL , Farquharson C . The functional co‐operativity of tissue‐nonspecific alkaline phosphatase (TNAP) and PHOSPHO1 during initiation of skeletal mineralization. Biochem Biophys Rep. 2015;4:196–201. 26457330 10.1016/j.bbrep.2015.09.013 PMC4594806

[34] Addison WN , Azari F , Sørensen ES , Kaartinen MT , McKee MD . Pyrophosphate inhibits mineralization of osteoblast cultures by binding to mineral, up‐regulating osteopontin, and inhibiting alkaline phosphatase activity. J Biol Chem. 2007;282(21):15872–83. 17383965 10.1074/jbc.M701116200

[35] Harmey D , Hessle L , Narisawa S , Johnson KA , Terkeltaub R , Millán JL . Concerted regulation of inorganic pyrophosphate and osteopontin by akp2, enpp1, and ank: an integrated model of the pathogenesis of mineralization disorders. Am J Pathol. 2004;164(4):1199–209. 15039209 10.1016/S0002-9440(10)63208-7 PMC1615351

[36] Johnson K , Goding J , Van Etten D , et al. Linked deficiencies in extracellular PPi and osteopontin mediate pathologic calcification associated with defective PC‐1 and ANK expression. J Bone Miner Res. 2003;18(6):994–1004. 12817751 10.1359/jbmr.2003.18.6.994

[37] Speer MY , McKee MD , Guldberg RE , et al. Inactivation of the osteopontin gene enhances vascular calcification of matrix Gla protein–deficient mice: evidence for osteopontin as an inducible inhibitor of vascular calcification in vivo. J Exp Med. 2002;196(8):1047–55. 12391016 10.1084/jem.20020911 PMC2194039

[38] Steitz SA , Speer MY , McKee MD , et al. Osteopontin inhibits mineral deposition and promotes regression of ectopic calcification. Am J Pathol. 2002;161(6):2035–46. 12466120 10.1016/S0002-9440(10)64482-3 PMC1850905

[39] Boskey A , Spevak L , Paschalis E , Doty S , McKee M . Osteopontin deficiency increases mineral content and mineral crystallinity in mouse bone. Calcif Tissue Int. 2002;71(2):145–54. 12073157 10.1007/s00223-001-1121-z

[40] Boskey AL , Christensen B , Taleb H , Sørensen ES . Post‐translational modification of osteopontin: effects on in vitro hydroxyapatite formation and growth. Biochem Biophys Res Commun. 2012;419(2):333–8. 22342723 10.1016/j.bbrc.2012.02.024 PMC3299831

[41] George A , Veis A . Phosphorylated proteins and control over apatite nucleation, crystal growth, and inhibition. Chem Rev. 2008;108(11):4670–93. 18831570 10.1021/cr0782729 PMC2748976

[42] Narisawa S , Yadav MC , Millán JL . In vivo overexpression of tissue‐nonspecific alkaline phosphatase increases skeletal mineralization and affects the phosphorylation status of osteopontin. J Bone Miner Res. 2013;28(7):1587–98. 23427088 10.1002/jbmr.1901 PMC3688694

[43] Yadav MC , Huesa C , Narisawa S , et al. Ablation of osteopontin improves the skeletal phenotype of Phospho1−/− mice. J Bone Miner Res. 2014;29(11):2369–81. 24825455 10.1002/jbmr.2281 PMC5247257

[44] McKee M , Yadav M , Foster B , Somerman M , Farquharson C , Millán J . Compounded PHOSPHO1/ALPL deficiencies reduce dentin mineralization. J Dent Res. 2013;92(8):721–7. 23694930 10.1177/0022034513490958 PMC3711567

[45] Zweifler L , Ao M , Yadav M , et al. Role of PHOSPHO1 in periodontal development and function. J Dent Res. 2016;95(7):742–51. 27016531 10.1177/0022034516640246 PMC4914864

[46] Ao M , Chavez M , Chu E , et al. Overlapping functions of bone sialoprotein and pyrophosphate regulators in directing cementogenesis. Bone. 2017;105:134–47. 28866368 10.1016/j.bone.2017.08.027 PMC5730356

[47] Pandya M , Rosene L , Farquharson C , Millán JL , Diekwisch TG . Intravesicular phosphatase PHOSPHO1 function in enamel mineralization and prism formation. Front Physiol. 2017;8:805. 29089903 10.3389/fphys.2017.00805 PMC5651051

[48] Kuzynski M , Goss M , Bottini M , et al. Dual role of the Trps1 transcription factor in dentin mineralization. J Biol Chem. 2014;289(40):27481–93. 25128529 10.1074/jbc.M114.550129 PMC4183789

[49] Napierala D , Sam K , Morello R , et al. Uncoupling of chondrocyte differentiation and perichondrial mineralization underlies the skeletal dysplasia in tricho‐rhino‐phalangeal syndrome. Hum Mol Genet. 2008;17(14):2244–54. 18424451 10.1093/hmg/ddn125 PMC2710999

[50] Fantauzzo KA , Tadin‐Strapps M , You Y , et al. A position effect on TRPS1 is associated with Ambras syndrome in humans and the Koala phenotype in mice. Hum Mol Genet. 2008;17(22):3539–51. 18713754 10.1093/hmg/ddn247 PMC2572698

[51] Momeni P , Glöckner G , Schmidt O , et al. Mutations in a new gene, encoding a zinc‐finger protein, cause tricho‐rhino‐phalangeal syndrome type I. Nat Genet. 2000;24(1):71. 10615131 10.1038/71717

[52] Kantaputra P , Miletich I , Lüdecke H‐J , et al. Tricho‐rhino‐phalangeal syndrome with supernumerary teeth. J Dent Res. 2008;87(11):1027–31. 18946009 10.1177/154405910808701102

[53] Cui L , Houston D , Farquharson C , MacRae V . Characterisation of matrix vesicles in skeletal and soft tissue mineralisation. Bone. 2016;87:147–58. 27072517 10.1016/j.bone.2016.04.007

[54] Anderson HC . Electron microscopic studies of induced cartilage development and calcification. J Cell Biol. 1967;35(1):81. 6061727 10.1083/jcb.35.1.81 PMC2107116

[55] Bonucci E . Fine structure of early cartilage calcification. J Ultrastruct Res. 1967;20(1–2):33–50. 4195919 10.1016/s0022-5320(67)80034-0

[56] Landis WJ , Paine MC , Glimcher MJ . Electron microscopic observations of bone tissue prepared anhydrously in organic solvents. J Ultrastruct Res. 1977;59(1):1–30. 66323 10.1016/s0022-5320(77)80025-7

[57] Anderson HC . Vesicles associated with calcification in the matrix of epiphyseal cartilage. J Cell Biol. 1969;41(1):59–72. 5775794 10.1083/jcb.41.1.59 PMC2107736

[58] Anderson HC , Cecil R , Sajdera SW . Calcification of rachitic rat cartilage in vitro by extracellular matrix vesicles. Am J Pathol. 1975;79(2):237. 1146961 PMC1912651

[59] Bonucci E . The locus of initial calcification in cartilage and bone. Clin Orthop Relat Res. 1971;78:108–39. 4939008 10.1097/00003086-197107000-00010

[60] Landis WJ . A study of calcification in the leg tendons from the domestic turkey. J Ultrastruct Mol Struct Res. 1986;94(3):217–38. 3027205 10.1016/0889-1605(86)90069-8

[61] Reynolds JL , Joannides AJ , Skepper JN , et al. Human vascular smooth muscle cells undergo vesicle‐mediated calcification in response to changes in extracellular calcium and phosphate concentrations: a potential mechanism for accelerated vascular calcification in ESRD. J Am Soc Nephrol. 2004;15(11):2857–67. 15504939 10.1097/01.ASN.0000141960.01035.28

[62] Kapustin AN , Shanahan CM . Calcium regulation of vascular smooth muscle cell–derived matrix vesicles. Trends Cardiovasc Med. 2012;22(5):133–7. 22902179 10.1016/j.tcm.2012.07.009

[63] Cui L , Rashdan NA , Zhu D , et al. End stage renal disease‐induced hypercalcemia may promote aortic valve calcification via Annexin VI enrichment of valve interstitial cell derived‐matrix vesicles. J Cell Physiol. 2017;232(11):2985–95. 28369848 10.1002/jcp.25935 PMC5575563

[64] Hasegawa T , Yamamoto T , Tsuchiya E , et al. Ultrastructural and biochemical aspects of matrix vesicle‐mediated mineralization. Jpn Dent Sci Rev. 2017;53(2):34–45. 28479934 10.1016/j.jdsr.2016.09.002 PMC5405202

[65] Bottini M , Mebarek S , Anderson KL , et al. Matrix vesicles from chondrocytes and osteoblasts: their biogenesis, properties, functions and biomimetic models. Biochim Biophys Acta Gen Subj. 2018;1862(3):532–46. 29108957 10.1016/j.bbagen.2017.11.005 PMC5801150

[66] Murshed M . Mechanism of bone mineralization. Cold Spring Harb Perspect Med. 2018;8:a031229. 29610149 10.1101/cshperspect.a031229 PMC6280711

[67] Anderson HC . Matrix vesicles and calcification. Curr Rheumatol Rep. 2003;5(3):222–6. 12744815 10.1007/s11926-003-0071-z

[68] Borg TK , Runyan RB , Wuthier RE . Correlation of freeze‐fracture and scanning electron microscopy of epiphyseal chondrocytes. Calcif Tissue Res. 1978;26(1):237–41. 750066 10.1007/BF02013264

[69] Anderson HC . Molecular biology of matrix vesicles. Clin Orthop Relat Res. 1995;314:266–80. 7634645

[70] Fedde KN . Human osteosarcoma cells spontaneously release matrix‐vesicle‐like structures with the capacity to mineralize. Bone Miner. 1992;17(2):145–51. 1611300 10.1016/0169-6009(92)90726-t

[71] Thouverey C , Strzelecka‐Kiliszek A , Balcerzak M , Buchet R , Pikula S . Matrix vesicles originate from apical membrane microvilli of mineralizing osteoblast‐like Saos‐2 cells. J Cell Biochem. 2009;106(1):127–38. 19009559 10.1002/jcb.21992

[72] Thouverey C , Bechkoff G , Pikula S , Buchet R . Inorganic pyrophosphate as a regulator of hydroxyapatite or calcium pyrophosphate dihydrate mineral deposition by matrix vesicles. Osteoarthritis Cartilage. 2009;17(1):64–72. 18603452 10.1016/j.joca.2008.05.020

[73] Shapiro IM , Golub EE , Chance B , et al. Linkage between energy status of perivascular cells and mineralization of the chick growth cartilage. Dev Biol. 1988;129(2):372–9. 3417044 10.1016/0012-1606(88)90384-3

[74] Wuthier RE , Lipscomb GF . Matrix vesicles: structure, composition, formation and function in calcification. Front Biosci. 2011;16:2812–902. 10.2741/3887 21622210

[75] Chaudhary SC , Kuzynski M , Bottini M , et al. Phosphate induces formation of matrix vesicles during odontoblast‐initiated mineralization in vitro. Matrix Biol. 2016;52:284–300. 26883946 10.1016/j.matbio.2016.02.003 PMC4875887

[76] Hessle L , Johnson KA , Anderson HC , et al. Tissue‐nonspecific alkaline phosphatase and plasma cell membrane glycoprotein‐1 are central antagonistic regulators of bone mineralization. Proc Natl Acad Sci. 2002;99(14):9445–9. 12082181 10.1073/pnas.142063399 PMC123160

[77] Stewart AJ , Roberts SJ , Seawright E , Davey MG , Fleming RH , Farquharson C . The presence of PHOSPHO1 in matrix vesicles and its developmental expression prior to skeletal mineralization. Bone. 2006;39(5):1000–7. 16837257 10.1016/j.bone.2006.05.014

[78] Millán JL . The role of phosphatases in the initiation of skeletal mineralization. Calcif Tissue Int. 2013;93(4):299–306. 23183786 10.1007/s00223-012-9672-8 PMC3594124

[79] Thouverey C , Malinowska A , Balcerzak M , et al. Proteomic characterization of biogenesis and functions of matrix vesicles released from mineralizing human osteoblast‐like cells. J Proteomics. 2011;74(7):1123–34. 21515422 10.1016/j.jprot.2011.04.005

[80] Ciancaglini P , Yadav MC , Simao AMS , et al. Kinetic analysis of substrate utilization by native and TNAP‐, NPP1‐, or PHOSPHO1‐deficient matrix vesicles. J Bone Miner Res. 2010;25(4):716–23. 19874193 10.1359/jbmr.091023 PMC3153326

[81] Yadav MC , Bottini M , Cory E , et al. Skeletal mineralization deficits and impaired biogenesis and function of chondrocyte‐derived matrix vesicles in Phospho1–/– and Phospho1/Pit1 double‐knockout mice. J Bone Miner Res. 2016;31(6):1275–86. 26773408 10.1002/jbmr.2790 PMC4891278

[82] Stewart AJ , Leong DT , Farquharson C . PLA2 and ENPP6 may act in concert to generate phosphocholine from the matrix vesicle membrane during skeletal mineralization. FASEB J. 2017;32(1):20–5. 28864658 10.1096/fj.201700521R

[83] Burke JE , Dennis EA . Phospholipase A2 structure/function, mechanism, and signaling. J Lipid Res. 2009;50(Suppl):S237–42. 19011112 10.1194/jlr.R800033-JLR200 PMC2674709

[84] Dennis EA , Cao J , Hsu Y‐H , Magrioti V , Kokotos G . Phospholipase A2 enzymes: physical structure, biological function, disease implication, chemical inhibition, and therapeutic intervention. Chem Rev. 2011;111(10):6130–85. 21910409 10.1021/cr200085w PMC3196595

[85] Murakami M , Taketomi Y , Miki Y , Sato H , Hirabayashi T , Yamamoto K . Recent progress in phospholipase A2 research: from cells to animals to humans. Prog Lipid Res. 2011;50(2):152–92. 21185866 10.1016/j.plipres.2010.12.001

[86] Wu LN , Genge BR , Kang MW , Arsenault AL , Wuthier RE . Changes in phospholipid extractability and composition accompany mineralization of chicken growth plate cartilage matrix vesicles. J Biol Chem. 2002;277(7):5126–33. 11714705 10.1074/jbc.M107899200

[87] Wuthier RE . Lipid composition of isolated epiphyseal cartilage cells, membranes and matrix vesicles. Biochim Biophys Acta. 1975;409(1):128–43. 1182191 10.1016/0005-2760(75)90087-9

[88] Fujino Y , Minamizaki T , Yoshioka H , Okada M , Yoshiko Y . Imaging and mapping of mouse bone using MALDI‐imaging mass spectrometry. Bone Rep. 2016;5:280–5. 28580397 10.1016/j.bonr.2016.09.004 PMC5440778

[89] Mebarek S , Abousalham A , Magne D , et al. Phospholipases of mineralization competent cells and matrix vesicles: roles in physiological and pathological mineralizations. Int J Mol Sci. 2013;14(3):5036–129. 23455471 10.3390/ijms14035036 PMC3634480

[90] Ramanadham S , Yarasheski KE , Silva MJ , et al. Age‐related changes in bone morphology are accelerated in group VIA phospholipase A2 (iPLA2β)‐null mice. Am J Pathol. 2008;172(4):868–81. 18349124 10.2353/ajpath.2008.070756 PMC2276416

[91] Stefan C , Jansen S , Bollen M . NPP‐type ectophosphodiesterases: unity in diversity. Trends Biochem Sci. 2005;30(10):542–50. 16125936 10.1016/j.tibs.2005.08.005

[92] Sakagami H , Aoki J , Natori Y , et al. Biochemical and molecular characterization of a novel choline‐specific glycerophosphodiester phosphodiesterase belonging to the nucleotide pyrophosphatase/phosphodiesterase family. J Biol Chem. 2005;280(24):23084–93. 15788404 10.1074/jbc.M413438200

[93] Morita J , Kato K , Mihara E , et al. Expression, purification, crystallization and preliminary X‐ray crystallographic analysis of Enpp6. Acta Crystallogr F Struct Biol Commun. 2014;70(6):794–9. 24915096 10.1107/S2053230X14008929 PMC4051540

[94] Khavandgar Z , Murshed M . Sphingolipid metabolism and its role in the skeletal tissues. Cell Mol Life Sci. 2015;72(5):959–69. 25424644 10.1007/s00018-014-1778-x PMC11114007

[95] Stoffel W , Jenke B , Blöck B , Zumbansen M , Koebke J . Neutral sphingomyelinase 2 (smpd3) in the control of postnatal growth and development. Proc Natl Acad Sci. 2005;102(12):4554–9. 15764706 10.1073/pnas.0406380102 PMC555473

[96] Stoffel W , Knifka J , Koebke J , et al. Neutral sphingomyelinase (SMPD3) deficiency causes a novel form of chondrodysplasia and dwarfism that is rescued by Col2A1‐driven smpd3 transgene expression. Am J Pathol. 2007;171(1):153–61. 17591962 10.2353/ajpath.2007.061285 PMC1941606

[97] Khavandgar Z , Poirier C , Clarke CJ , et al. A cell‐autonomous requirement for neutral sphingomyelinase 2 in bone mineralization. J Cell Biol. 2011;194(2):277–89. 21788370 10.1083/jcb.201102051 PMC3144407

[98] Li J , Manickam G , Ray S , Yasuda H , Moffatt P , Murshed M . Smpd3 expression in both chondrocytes and osteoblasts is required for normal endochondral bone development. Mol Cell Biol. 2016;36(17):2282–99. 27325675 10.1128/MCB.01077-15 PMC4985927

[99] Khavandgar Z , Alebrahim S , Eimar H , Tamimi F , McKee M , Murshed M . Local regulation of tooth mineralization by sphingomyelin phosphodiesterase 3. J Dent Res. 2013;92(4):358–64. 23428435 10.1177/0022034513478429

[100] Manickam G , Moffatt P , Murshed M . Role of SMPD3 during bone fracture healing and regulation of its expression. Mol Cell Biol. 2019;39(4):e00370‐18. 30530524 10.1128/MCB.00370-18 PMC6362318

[101] Wu G , Aoyama C , Young SG , Vance DE . Early embryonic lethality caused by disruption of the gene for choline kinase α, the first enzyme in phosphatidylcholine biosynthesis. J Biol Chem. 2008;283(3):1456–62. 18029352 10.1074/jbc.M708766200 PMC3033886

[102] Li Z , Wu G , Sher RB , et al. Choline kinase beta is required for normal endochondral bone formation. Biochim Biophys Acta Gen Subj. 2014;1840(7):2112–22. 10.1016/j.bbagen.2014.03.008 PMC4143985 24637075

[103] Li Z , Wu G , van der Veen JN , Hermansson M , Vance DE . Phosphatidylcholine metabolism and choline kinase in human osteoblasts. Biochim Biophys Acta Mol Cell Biol Lipids. 2014;1841(6):859–67. 10.1016/j.bbalip.2014.02.004 24583375

[104] Compston JE . Sex steroids and bone. Physiol Rev. 2001;81(1):419–47. 11152762 10.1152/physrev.2001.81.1.419

[105] Frenkel B , Hong A , Baniwal SK , et al. Regulation of adult bone turnover by sex steroids. J Cell Physiol. 2010;224(2):305–10. 20432458 10.1002/jcp.22159 PMC5770230

[106] Riggs BL , Khosla S , Melton LJ 3rd . Sex steroids and the construction and conservation of the adult skeleton. Endocr Rev. 2002;23(3):279–302. 12050121 10.1210/edrv.23.3.0465

[107] Thacher TD , Clarke BL . Vitamin D insufficiency. Mayo Clin Proc. 2011;86(1):50–60. 21193656 10.4065/mcp.2010.0567 PMC3012634

[108] Yamamoto Y , Yoshizawa T , Fukuda T , et al. Vitamin D receptor in osteoblasts is a negative regulator of bone mass control. Endocrinology. 2013;154(3):1008–20. 23389957 10.1210/en.2012-1542

[109] Priemel M , von Domarus C , Klatte TO , et al. Bone mineralization defects and vitamin D deficiency: histomorphometric analysis of iliac crest bone biopsies and circulating 25‐hydroxyvitamin D in 675 patients. J Bone Miner Res. 2010;25(2):305–12. 19594303 10.1359/jbmr.090728

[110] Compston JE . Skeletal actions of intermittent parathyroid hormone: effects on bone remodelling and structure. Bone. 2007;40(6):1447–52. 17045858 10.1016/j.bone.2006.09.008

[111] Jiang Y , Zhao JJ , Mitlak BH , Wang O , Genant HK , Eriksen EF . Recombinant human parathyroid hormone (1–34)[teriparatide] improves both cortical and cancellous bone structure. J Bone Miner Res. 2003;18(11):1932–41. 14606504 10.1359/jbmr.2003.18.11.1932

[112] Hodsman AB , Bauer DC , Dempster DW , et al. Parathyroid hormone and teriparatide for the treatment of osteoporosis: a review of the evidence and suggested guidelines for its use. Endocr Rev. 2005;26(5):688–703. 15769903 10.1210/er.2004-0006

[113] John H. C. S. , Meyer MB , Benkusky NA , et al. The parathyroid hormone‐regulated transcriptome in osteocytes: parallel actions with 1, 25‐dihydroxyvitamin D3 to oppose gene expression changes during differentiation and to promote mature cell function. Bone. 2015;72:81–91. 25460572 10.1016/j.bone.2014.11.010 PMC4285334

[114] Houston D , Myers K , MacRae V , Staines K , Farquharson C . The expression of PHOSPHO1, nSMase2 and TNAP is coordinately regulated by continuous PTH exposure in mineralising osteoblast cultures. Calcif Tissue Int. 2016;99(5):510–24. 27444010 10.1007/s00223-016-0176-9 PMC5055575

[115] Allan EH , Häusler KD , Wei T , et al. EphrinB2 regulation by PTH and PTHrP revealed by molecular profiling in differentiating osteoblasts. J Bone Miner Res. 2008;23(8):1170–81. 18627264 10.1359/jbmr.080324

[116] Gooi J , Pompolo S , Karsdal M , et al. Calcitonin impairs the anabolic effect of PTH in young rats and stimulates expression of sclerostin by osteocytes. Bone. 2010;46(6):1486–97. 20188226 10.1016/j.bone.2010.02.018

[117] Eriksen EF . Primary hyperparathyroidism: lessons from bone histomorphometry. J Bone Miner Res. 2002;17 Suppl 2:N95–7. 12412784

[118] Locklin RM , Khosla S , Turner RT , Riggs BL . Mediators of the biphasic responses of bone to intermittent and continuously administered parathyroid hormone. J Cell Biochem. 2003;89(1):180–90. 12682918 10.1002/jcb.10490

[119] Ma YL , Cain RL , Halladay DL , et al. Catabolic effects of continuous human PTH (1–38) in vivo is associated with sustained stimulation of RANKL and inhibition of osteoprotegerin and gene‐associated bone formation. Endocrinology. 2001;142(9):4047–54. 11517184 10.1210/endo.142.9.8356

[120] Krishnan V , Moore TL , Ma YL , et al. Parathyroid hormone bone anabolic action requires Cbfa1/Runx2‐dependent signaling. Mol Endocrinol. 2003;17(3):423–35. 12554794 10.1210/me.2002-0225

[121] Nishimura R , Wakabayashi M , Hata K , et al. Osterix regulates calcification and degradation of chondrogenic matrices through matrix metalloproteinase 13 (MMP13) expression in association with transcription factor Runx2 during endochondral ossification. J Biol Chem. 2012;287(40):33179–90. 22869368 10.1074/jbc.M111.337063 PMC3460424

[122] Kiffer‐Moreira T , Yadav MC , Zhu D , et al. Pharmacological inhibition of PHOSPHO1 suppresses vascular smooth muscle cell calcification. J Bone Miner Res. 2013;28(1):81–91. 22887744 10.1002/jbmr.1733 PMC3562655

[123] Tan L , Wang Z , Li Y . Rabbit models provide insights into bone formation related biological process in atherosclerotic vascular calcification. Biochem Biophys Res Commun. 2018;496(4):1369–75. 29421657 10.1016/j.bbrc.2018.02.035

[124] Hortells L , Sosa C , Guillén N , Lucea S , Millán Á. , Sorribas V . Identifying early pathogenic events during vascular calcification in uremic rats. Kidney Int. 2017;92(6):1384–94. 28844316 10.1016/j.kint.2017.06.019

[125] Bobryshev YV , Orekhov AN , Sobenin I , Chistiakov DA . Role of bone‐type tissue‐nonspecific alkaline phosphatase and PHOSPO1 in vascular calcification. Curr Pharm Des. 2014;20(37):5821–8. 24533943 10.2174/1381612820666140212193011

[126] Bertazzo S , Gentleman E . Aortic valve calcification: a bone of contention. Eur Heart J. 2017;38(16):1189–93. 26994153 10.1093/eurheartj/ehw071 PMC5400053

[127] Bertazzo S , Gentleman E , Cloyd KL , Chester AH , Yacoub MH , Stevens MM . Nano‐analytical electron microscopy reveals fundamental insights into human cardiovascular tissue calcification. Nat Mater. 2013;12(6):576. 23603848 10.1038/nmat3627 PMC5833942

[128] Hutcheson JD , Goettsch C , Bertazzo S , et al. Genesis and growth of extracellular‐vesicle‐derived microcalcification in atherosclerotic plaques. Nat Mater. 2016;15(3):335. 26752654 10.1038/nmat4519 PMC4767675

[129] van Engeland NC , Bertazzo S , Sarathchandra P , et al. Aortic calcified particles modulate valvular endothelial and interstitial cells. Cardiovasc Pathol. 2017;28:36–45. 28319833 10.1016/j.carpath.2017.02.006

[130] Li B , Aspden RM . Mechanical and material properties of the subchondral bone plate from the femoral head of patients with osteoarthritis or osteoporosis. Ann Rheum Dis. 1997;56(4):247–54. 9165997 10.1136/ard.56.4.247 PMC1752348

[131] Chen L , Fink T , Zhang X‐Y , Ebbesen P , Zachar V . Quantitative transcriptional profiling of ATDC5 mouse progenitor cells during chondrogenesis. Differentiation. 2005;73(7):350–63. 16219039 10.1111/j.1432-0436.2005.00038.x

[132] Staines KA , Brain F , Javaheri B , et al. Hypomineralisation drives joint instability and osteoarthritis in mice. In: Committee BRS, editor. Bone Research Society Annual Meeting 2016; Liverpool, UK: Frontiers in Bone Research; 2016. p. 39.

[133] Kosinska MK , Liebisch G , Lochnit G , et al. A lipidomic study of phospholipid classes and species in human synovial fluid. Arthritis Rheum. 2013;65(9):2323–33. 23784884 10.1002/art.38053

[134] Pruzanski W , Bogoch E , Wloch M , Vadas P . The role of phospholipase A2 in the physiopathology of osteoarthritis. J Rheumatol Suppl. 1991;27:117–9. 2027108

[135] Leistad L , Feuerherm A , Faxvaag A , Johansen B . Multiple phospholipase A2 enzymes participate in the inflammatory process in osteoarthritic cartilage. Scand J Rheumatol. 2011;40(4):308–16. 21417548 10.3109/03009742.2010.547872

[136] Fuchs B , Schiller J , Wagner U , Häntzschel H , Arnold K . The phosphatidylcholine/lysophosphatidylcholine ratio in human plasma is an indicator of the severity of rheumatoid arthritis: investigations by 31P NMR and MALDI‐TOF MS. Clin Biochem. 2005;38(10):925–33. 16043165 10.1016/j.clinbiochem.2005.06.006

[137] van der Veen JN , Kennelly JP , Wan S , Vance JE , Vance DE , Jacobs RL . The critical role of phosphatidylcholine and phosphatidylethanolamine metabolism in health and disease. Biochim Biophys Acta Biomembr. 2017;1859(9 Pt B):1558–72. 28411170 10.1016/j.bbamem.2017.04.006

[138] Cole LK , Vance JE , Vance DE . Phosphatidylcholine biosynthesis and lipoprotein metabolism. Biochim Biophys Acta Mol Cell Biol Lipids. 2012;1821(5):754–61. 10.1016/j.bbalip.2011.09.009 21979151

[139] Exton J . Phosphatidylcholine breakdown and signal transduction. Biochim Biophys Acta. 1994;1212(1):26–42. 8155724 10.1016/0005-2760(94)90186-4

[140] Exton J . Signaling through phosphatidylcholine breakdown. J Biol Chem. 1990;265(1):1–4. 2104616

[141] Li Z , Vance DE . Thematic review series: glycerolipids. Phosphatidylcholine and choline homeostasis. J Lipid Res. 2008;49(6):1187–94. 18204095 10.1194/jlr.R700019-JLR200

[142] Alves da Silva AV , de Castro Oliveira SB , Di Rienzi SC , et al. Murine methyl donor deficiency impairs early growth in association with dysmorphic small intestinal crypts and reduced gut microbial community diversity. Curr Dev Nutr. 2018;3(1):nzy070.

[143] Westra H. ‐J. , Peters MJ , Esko T , et al. Systematic identification of trans eQTLs as putative drivers of known disease associations. Nat Genet. 2013;45(10):1238. 24013639 10.1038/ng.2756 PMC3991562

[144] Gautier E‐F , Ducamp S , Leduc M , et al. Comprehensive proteomic analysis of human erythropoiesis. Cell Rep. 2016;16(5):1470–84. 27452463 10.1016/j.celrep.2016.06.085 PMC5274717

[145] Huang N‐J , Lin Y‐C , Lin C‐Y , et al. Enhanced phosphocholine metabolism is essential for terminal erythropoiesis. Blood. 2018;131(26):2955–66. 29712634 10.1182/blood-2018-03-838516 PMC6024642

[146] Chambers JC , Loh M , Lehne B , et al. Epigenome‐wide association of DNA methylation markers in peripheral blood from Indian Asians and Europeans with incident type 2 diabetes: a nested case‐control study. Lancet Diabetes Endocrinol. 2015;3(7):526–34. 26095709 10.1016/S2213-8587(15)00127-8 PMC4724884

[147] Dayeh T , Tuomi T , Almgren P , et al. DNA methylation of loci within ABCG1 and PHOSPHO1 in blood DNA is associated with future type 2 diabetes risk. Epigenetics. 2016;11(7):482–8. 27148772 10.1080/15592294.2016.1178418 PMC4939923

[148] Willmer T , Johnson R , Louw J , Pheiffer C . Blood based DNA methylation biomarkers for type 2 diabetes: potential for clinical applications. Front Endocrinol. 2018;9:744. 10.3389/fendo.2018.00744 PMC6288427 30564199

[149] Sayols‐Baixeras S , Subirana I , Lluis‐Ganella C , et al. Identification and validation of seven new loci showing differential DNA methylation related to serum lipid profile: an epigenome‐wide approach. The REGICOR study. Hum Mol Genet. 2016;25(20):4556–65. 28173150 10.1093/hmg/ddw285 PMC6284258

[150] Wu Y , Duan H , Tian X , et al. Genetics of obesity traits: a bivariate genome‐wide association analysis. Front Genet. 2018;9:179. 29868124 10.3389/fgene.2018.00179 PMC5964872

[151] Wang H , Liu L , Lin JZ , Aprahamian TR , Farmer SR . Browning of white adipose tissue with roscovitine induces a distinct population of UCP1+ adipocytes. Cell Metab. 2016;24(6):835–47. 27974179 10.1016/j.cmet.2016.10.005 PMC6674884

[152] Lee J , Choi J , Aja S , Scafidi S , Wolfgang MJ . Loss of adipose fatty acid oxidation does not potentiate obesity at thermoneutrality. Cell Rep. 2016;14(6):1308–16. 26854223 10.1016/j.celrep.2016.01.029 PMC4758873

[153] Kazak L , Chouchani ET , Jedrychowski MP , et al. A creatine‐driven substrate cycle enhances energy expenditure and thermogenesis in beige fat. Cell. 2015;163(3):643–55. 26496606 10.1016/j.cell.2015.09.035 PMC4656041

[154] Bertholet AM , Kazak L , Chouchani ET , et al. Mitochondrial patch clamp of beige adipocytes reveals UCP1‐positive and UCP1‐negative cells both exhibiting futile creatine cycling. Cell Metab. 2017;25(4):811–22.e4. 28380374 10.1016/j.cmet.2017.03.002 PMC5448977

[155] Delomenède M , Buchet R , Mebarek S . Lansoprazole is an uncompetitive inhibitor of tissue‐nonspecific alkaline phosphatase. Acta Biochimica Polonica. 2009;56(2):301. 19543559

[156] Bravo Y , Teriete P , Dhanya R‐P , et al. Design, synthesis and evaluation of benzoisothiazolones as selective inhibitors of PHOSPHO1. Bioorg Med Chem Lett. 2014;24(17):4308–11. 25124115 10.1016/j.bmcl.2014.07.013 PMC4170737

